# Serum hepatokines in dairy cows: periparturient variation and changes in energy-related metabolic disorders

**DOI:** 10.1186/s12917-018-1560-7

**Published:** 2018-08-13

**Authors:** Jianguo Wang, Xiaoyan Zhu, Guanghui She, Yezi Kong, Yazhou Guo, Zhe Wang, Guowen Liu, Baoyu Zhao

**Affiliations:** 1 College of Veterinary Medicine, Northwest A&F University, Yangling, 712100 Shaanxi China; 2 UMR 1195 Inserm and University Paris-Saclay, Kremlin-Bicêtre, France; 3 College of Veterinary Medicine, Jilin University, Changchun, 130062 Jilin China

**Keywords:** Dairy cow, Peripartum period, Angiopoietin-like protein 4, Fibroblast growth factor 21, Energy metabolism disorder

## Abstract

**Background:**

During peripartum period, dairy cows are highly susceptible to energy metabolism disorders such as fatty liver and ketosis. Angiopoietin-like protein 4 (ANGPTL4) and fibroblast growth factor 21 (FGF21), known as hepatokines, play important roles in lipid metabolism. The purposes of our study were to evaluate variations of serum ANGPTL4 and FGF21 concentrations in periparturient dairy cows and changes in these serum analyte concentrations of energy-related metabolic disorders in early lactation dairy cows.

This study was divided into two experiments. *Experiment I:* Blood parameters were measured in healthy periparturient Holstein cows from 4 wk antepartum to 4 wk postpartum (*n* = 219). In this experiment, weekly blood samples were obtained from 4 wk before the expected calving date through 4 wk after calving. *Experiment II:* Blood parameters were measured in healthy cows (*n* = 30) and cows with clinical ketosis (*n* = 29) and fatty liver (*n* = 25) within the first 4 wk of lactation. In the present study, all blood samples were collected from the coccygeal vein in the early morning before feeding.

**Results:**

Serum ANGPTL4 and FGF21 concentrations peaked at parturition, and declined rapidly over the following 2 wk Serum ANGPTL4 and FGF21 concentrations were positively correlated with serum non-esterified fatty acids (NEFA) concentration (*r* = 0.856, *P* = 003; *r* = 0.848, *P* = 0.004, respectively). Cows with clinical ketosis and fatty liver had significantly higher serum ANGPTL4 and FGF21 concentrations than healthy cows (*P* < 0.01).

**Conclusion:**

Serum ANGPTL4 and FGF21 concentrations were elevated during peripartum period, suggesting that energy balance changes that were associated with parturition contributed significantly to these effects. Although FGF21 and ANGPTL4 could play important roles in the adaptation of energy metabolism, they may be involved in the pathological processes of energy metabolism disorders of dairy cows in the peripartum period.

## Background

The periparturient period, from 4 wk antepartum to 4 wk postpartum, is vitally important to the health status and the reproductive performance of dairy cows. During this period, dairy cows experience a dramatic transition from the pregnant, non-lactating state to the non-pregnant, lactating state [1]. Postpartum milk production leads to a large increase in energy requirements that cannot be met solely through dietary intake. As a result, dairy cows enter a negative energy balance (NEB) that is induced by nutrient prioritization towards the mammary gland [2, 3]. Under NEB conditions, cows start to mobilize their body reserves from adipose tissue, resulting in elevated plasma concentrations of non-esterified fatty acids (NEFA). Although NEFA can be used directly as a fuel source by various tissues such as muscle, and used for milk fat synthesis by the mammary gland.

Liver removes 15 to 20% of NEFA from the bloodstream, in which NEFA can undergo mitochondrial β-oxidation to produce energy [4]. The liver may receive up to 38% of cardiac output, therefore it is susceptible to an oversupply of NEFA when arterial concentrations are elevated [5]. Excessive NEFA will be incompletely oxidized by the liver to generate ketone bodies or re-esterified to form triacylglycerols (TAG), thereby potentially resulting in the development of metabolic disorders associated with NEB, such as ketosis and fatty liver [6]. These disorders are associated with increased veterinary costs, longer calving intervals, decreased milk production, and decreased average lifetime of cows, causing huge economic losses for the dairy industry [6, 7].

Angiopoietin-like protein 4 (ANGPTL4), also known as the fasting-induced adipose factor (FIAF), and fibroblast growth factor 21 (FGF21) have been identified as hepatokines, which are predominantly expressed in the liver and adipose tissue [8, 9]. In dairy cows, most circulating ANGPTL4 and FGF21 originate from the liver [10, 11]. ANGPTL4 is a secreted glycoprotein that can inhibit lipoprotein lipase (LPL) activity and stimulate lipolysis, thereby regulating systemic energy and lipid metabolism. FGF21 has also been identified as a hormonal factor involved in the regulation of metabolic adaptations, such as promotion of hepatic lipid oxidation and ketogenesis, during energy deprivation [12]. Nutritional status can drive expression and secretion of ANGPTL4 and FGF21 in human, mouse and bovine subjects [13]. Under fasting conditions, expression levels of ANGPTL4 and FGF21 are strongly upregulated in the liver and white adipose tissue [9, 14]. Moreover, our previous report demonstrated that NEFA can promote ANGPTL4 and FGF21 expression and secretion in bovine hepatocytes cultured in vitro [15, 16]. However, the in vivo kinetics of ANGPTL4 and FGF21 are not entirely clear in dairy cows. In addition to our interest in whether ANGPTL4 and FGF21 participate in the pathological process of energy metabolic disorders in cows, we hypothesized that the serum concentration of these two hepatokines in dairy cows with ketosis and fatty liver are different from the contemporary healthy cows. Therefore, the purposes of this study were to evaluate variations in serum concentrations of ANGPTL4 and FGF21 in periparturient dairy cows, to examine correlations between serum NEFA and β-hydroxybutyrate (BHBA) concentrations and ANGPTL4 and FGF21 concentrations in the serum of the experimental cows, and to compare the concentrations of hepatokines in serum samples from cows with spontaneous ketosis and fatty liver, and healthy cows.

## Results

### Changes of serum hepatokines in periparturient dairy cows

In the first part of the study, 310 nonlactating multiparous Chinese Holstein dairy cows in late gestation were housed in a free-stall barn and included in the study from 4 wk before the anticipated calving date through 4 wk after parturition. The cows were 3–6 years old with 1–4 parities and had similar initial body condition scores (BCS, range 3.0–3.5, on a scale of 1–5). During the experiment, 65 cows had subclinical or clinical hypocalcemia, one cow had twins, and 18 cows calved much earlier and 7 cows calved much later than their anticipated calving dates. Ninety-one animals were eventually excluded from the experiment. Finally, 219 cows were chosen.

**Fig. 1:**
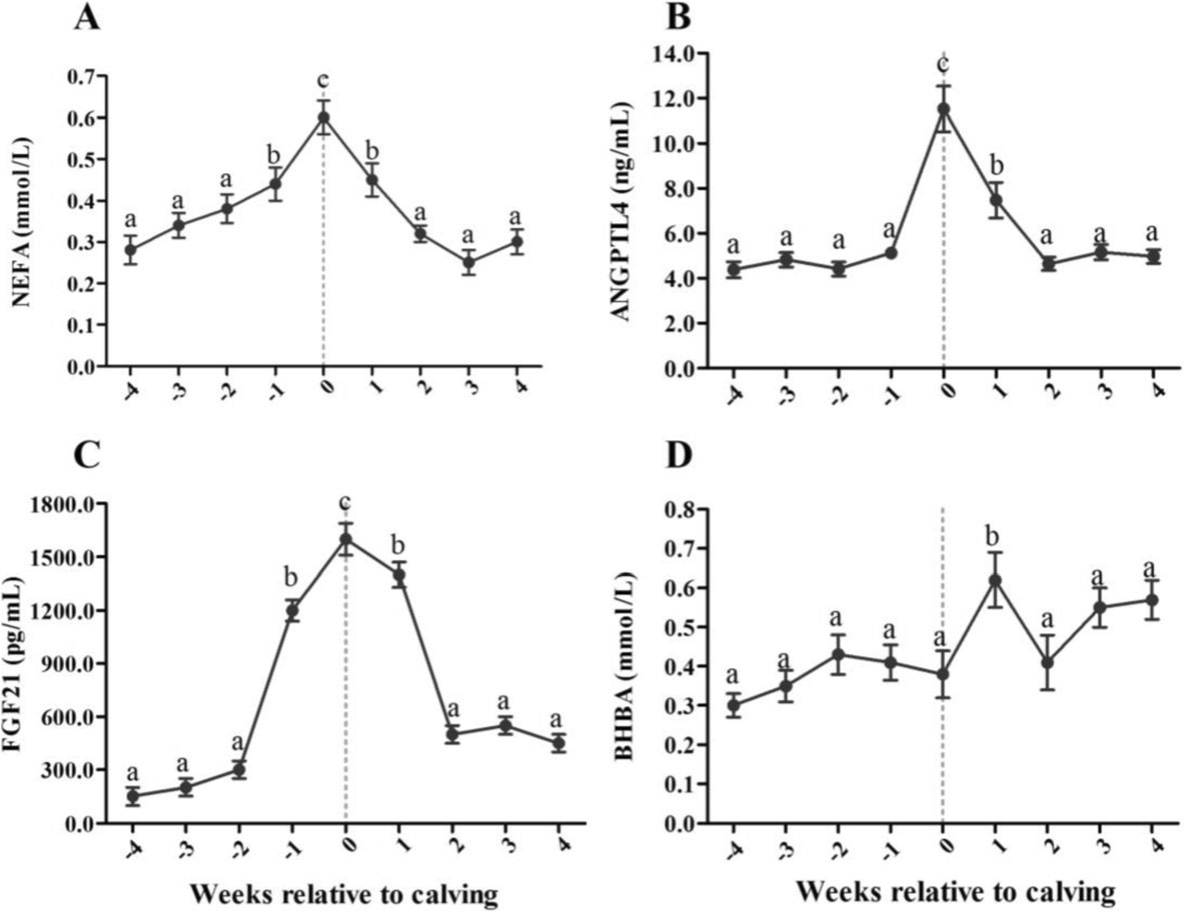
The variation of serum ANGPTL4, FGF21, NEFA and BHBA concentrations in perparturient dairy cows (*n* = 219). Serum NEFA (**a**), ANGPTL4 (**b**) and FGF21 (**c**) concentrations peaked on the day of parturition and were significantly higher during parturition than in late pregnancy (2 to 4 wk antepartum) and early lactation (2 to 4 wk postpartum). Serum BHBA (**d**) concentration was significantly higher at 1 wk postpartum than during late pregnancy (1 to 4 wk antepartum) and early lactation (2 to 4 wk postpartum). Results are presented as means ± SE and were analyzed using repeated-measures analysis of variance (ANOVA) followed by Bonferroni’s multiple comparison test. The neighboring lowercase letters show significant differences at *P* < 0.05, while the divided lowercase letters show very significant differences at *P* < 0.01. The same lowercase letters show no significant differences at *P* > 0.05

As shown in Fig. 1, similar to the NEFA concentration, serum ANGPTL4 and FGF21 concentrations peaked on the day of parturition and were significantly higher during parturition than in late pregnancy (2 to 4 wk antepartum) and early lactation (2 to 4 wk postpartum) (*P* < 0.01). Serum BHBA concentration was significantly higher at 1 wk postpartum than during late pregnancy (1 to 4 wk antepartum) and early lactation (2 to 4 wk postpartum) (*P* < 0.05).

Serum NEFA concentration was positively correlated with serum concentrations of ANGPTL4 (*r* = 0.856, *P* = 0.003) and FGF21 (*r* = 0.848, *P* = 0.004) in experimental cows during the periparturient period. However, serum BHBA concentration did not appear to be associated with serum concentrations of ANGPTL4 (*r* = 0.066, *P* = 0.866) and FGF21 (*r* = 0.295, *P* = 0.442) in experimental cows.

### Serum analytes in cows with energy-related metabolic disorders

**Fig. 2:**
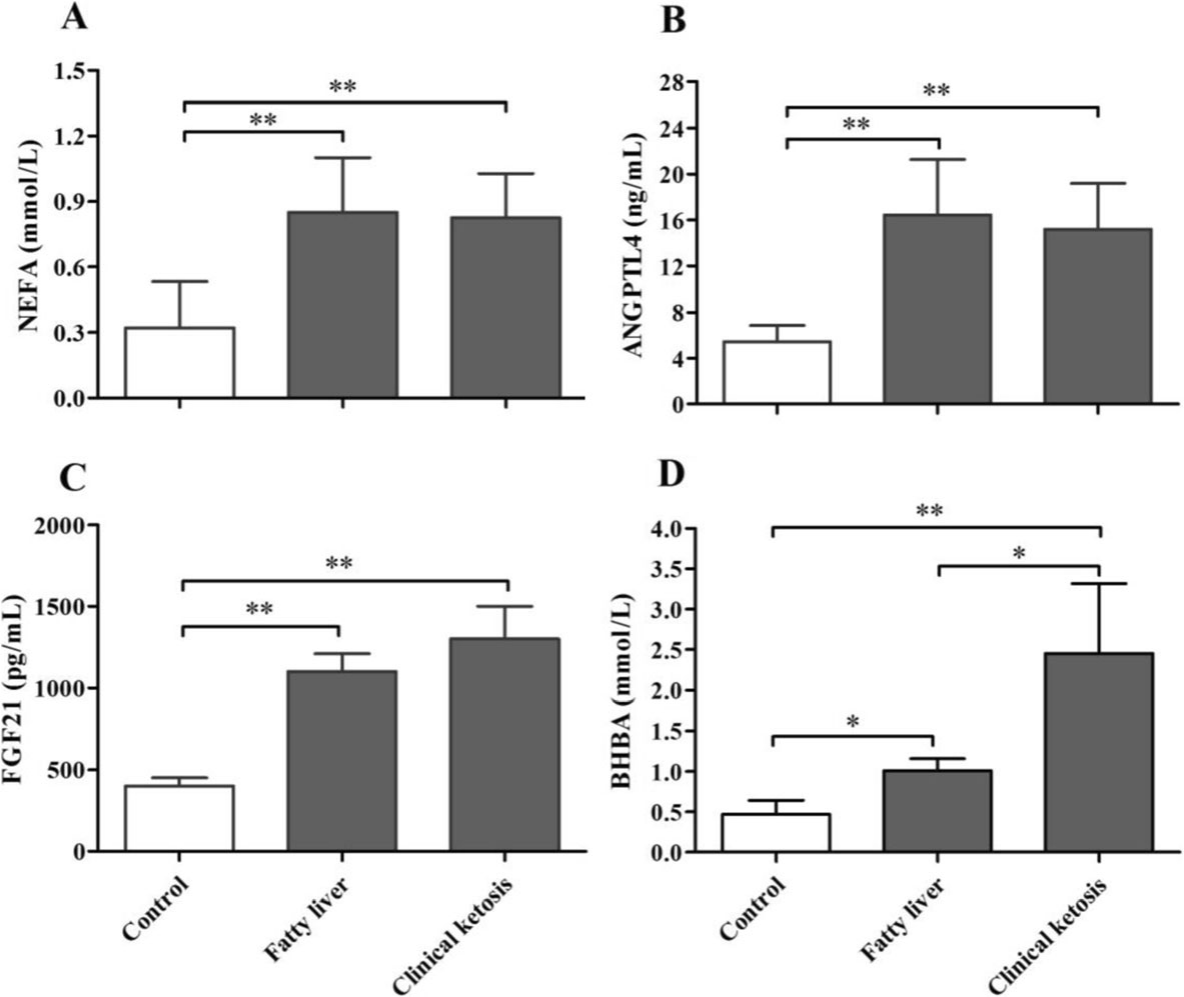
Levels of ANGPTL4, FGF21, NEFA and BHBA were analyzed in the serum of healthy control cows (*n* = 30) and those affected by clinical ketosis (*n* = 29) and fatty liver (*n* = 25). In the serum, concentrations of NEFA (**a**), ANGPTL4 (**b**), FGF21 (**c**) and BHBA (**d**) were higher in the cows with fatty liver and clinical ketosis than in the healthy control cows. Results are presented as means ± SE and were analyzed by one-way ANOVA followed by Dunnett’s multiple comparisons test. *P < 0.05, **P < 0.01 when compared to the healthy control cows. The comparisons between cows with clinical ketosis and fatty liver were analyzed by unpaired *t*-test. No significant differences in the serum concentrations of NEFA, ANGPTL4 and FGF21 were measured between cows with clinical ketosis and fatty liver (*P* > 0.05). As the basis of ketosis diagnosis, the serum BHBA levels were higher (*P* < 0.05) in the cows with clinical ketosis (2.45 ± 0.71) than in the cows with fatty liver (1.01 ± 0.15)

Compared with healthy cows, cows with fatty liver and clinical ketosis exhibited significant higher serum concentrations of NEFA, ANGPTL4 and FGF21 (*P* < 0.01, Fig. 2). In addition, BHBA concentrations were significantly higher in clinically ketotic cows (*P* < 0.01) and cows with fatty liver (*P* < 0.05) than in the control group (Fig. 2).

The activities of enzymes aspartate aminotransferase (AST), alanine aminotransferase (ALT), lactic dehydrogenase (LDH) and alkaline phosphatase (ALP), and the concentrations of two proinflammatory factors, tumor necrosis factor-α (TNF-α) and interleukin-6 (IL-6), in the serum of cows with normal and energy-related metabolic disorders are shown in Table 1. There were significant increases in the activities of AST, ALT and LDH, and the concentrations of TNF-α and IL-6 in the serum of cows with fatty liver (*P* < 0.01) than in the control group. The enzymes AST and LDH were significantly greater in the serum of ketotic cows (*P* < 0.01) than in the control group. ALT activity and IL-6 level were raised, while TNF-α was decreased in the serum of cows with ketosis although not statistically significant. There were no significant differences in the activity of serum ALP of cows with clinical ketosis and fatty liver when compared with healthy cows (*P* > 0.05).

**Table 1:** Selected metabolic parameters in the experimental and healthy control groups (means ± SE)

Parameter	Healthy (*n* = 30)	Clinical Ketosis (*n* = 29)	Fatty liver (*n* = 25)
AST (U/L)	42.15 ± 3.12	160.28 ± 15.79**	348.13 ± 25.5**
ALT (U/L)	19.21 ± 0.45	22.29 ± 0.93	74.30 ± 2.15**
LDH (U/L)	862.31 ± 22.21	1012.73 ± 55.15**	1048.60 ± 63.67**
ALP (U/L)	51.33 ± 3.85	53.10 ± 2.18	49.96 ± 3.39
TNF-α (ng/L)	206.97 ± 35.39	170.65 ± 15.10	1551.12 ± 102.37**
IL-6 (ng/L)	6.42 ± 0.60	7.13 ± 1.95	59.23 ± 10.34**

**P < 0.01 versus healthy cows

## Discussion

The periparturient period is challenging for both cows and producers due to the rapid increase in energy demands required to support milk production after parturition [17]. During the early lactation period, dietary intake alone cannot meet the enormous energy demands associated with milk production, thereby resulting in NEB, which then leads to the extensive mobilization of fatty acid stored in adipose tissue and causes marked elevations in blood NEFA concentrations. Therefore, serum concentrations of NEFA reflect the magnitude of the mobilization of fat from storage and mirror levels of dry matter intake (DMI) [18]. For exactly these reasons, serum NEFA is considered to be one of the most important indicators of energy balance status [19]. The present study showed that serum NEFA peaked on the day of parturition and declined rapidly over the following 1 wk This result is consistent with previous findings [20, 21].

Peroxisome proliferator-activated receptor α (PPARα) is one of the key transcription factors for regulating lipid metabolism [22]. Research has shown that the NEFA ligand can lead to the activation of PPARα in the liver during early lactation, thus promoting expression of its target genes [23, 24]. FGF21 and ANGPTL4 have been shown to be downstream targets of PPARα. Schlegel and colleagues observed that FGF21 expression was strongly upregulated during the transition period [12]. ANGPTL4 expression and secretion can be driven by the nutritional status of humans and rodents [25, 26], with the plasma ANGPTL4 concentration increasing with fasting and decreasing with high-fat feeding [9]. In the present study, we showed that serum FGF21 and ANGPTL4 concentrations were significantly increased during parturition. The serum NEFA concentration was significantly and positively correlated with concentrations of FGF21 and ANGPTL4 in experimental cows during the periparturient period. Some researchers have identified FGF21 and ANGPTL4 as hepatokines that are markedly responsive to energy balance changes in lactating dairy cows [27, 28]. Moreover, there is evidence that NEFA can trigger hepatic FGF21 and ANGPTL4 upregulation and production through the activation of PPARα in nonruminants. The effects of these fatty acids may explain the trends in serum FGF21 and ANGPTL4 concentrations that varied consistently with NEFA concentration trends in dairy cattle during the periparturient period.

It is worth noting that hepatokines may also influence lipid mobilization in a manner that is dependent on the levels of other hormones, such as growth hormone (GH), adiponectin and insulin. Khan and coworkers indicated that the activation of FGF21 could inhibit or block local GH signaling, and when in circulation, FGF21 can reduce adipose tissue insulin sensitivity and contribute to lipolysis in dairy cows [29]. The reduced insulin sensitivity of adipose tissue during early lactation might be associated with the decreased adiponectin sensitivity in adipose tissue of dairy cows after calving [30]. Adiponectin, a hormone secreted by adipocytes, plays crucial roles in regulating glucose and lipid metabolism and insulin sensitivity. Lin et al. [31] and Holland et al. [32] showed that in mice, FGF21 is a potent regulator of adiponectin secretion and critically depends on adiponectin to exert its energy glycemic and insulin sensitizing effects. In the present study, we found that serum FGF21 concentrations were significantly increased during parturition. In cattle, adiponectin mRNA is highly expressed in adipose tissues. However, adiponectin mRNA expression in adipose tissue is lower in lactating cows than in non-lactating cows [33, 34]. Although these results may be specific in ruminants, the details of these mechanisms are still unclear. There are several possible mechanisms that could account for the paradoxical dissociation between serum FGF21 levels and adiponectin expression in dairy cows during the periparturient period. First, FGF21 is not the only regulator of adiponectin production. Many factors that negatively regulate adiponectin expression and secretion, such as endoplasmic reticulum stress (ERS), and oxidative stress, are progressively increased during the transition period [35, 36]. Therefore, elevated FGF21 is not sufficient to counteract the effects of these negative regulators on adiponectin production under this circumstance. Second, it has been reported the decreases of adiponectin mRNA levels in adipose tissues and serum adiponectin concentrations induced by the NEB during lactation [37]. Therefore, it is likely that the ability of FGF21 to induce adipocyte production of adiponectin is diminished due to severe NEB during parturition. Some studies have shown that in non-ruminant animals, ANGPTL4 exerts distinct effects on lipid and glucose metabolism [25]. Activation of ANGPTL4 contributes to lipolysis by inhibiting LPL activity in adipose tissue, whereas the genetic and viral-mediated overexpression of ANGPTL4 potently decreases the blood glucose concentration by suppressing basal glucose output and inhibiting hepatic glucose production in mice [38]. The inhibitory effect of ANGPTL4 on hepatic glucose production is similar to adiponectin, although they might act through distinct pathways [25]. In addition, very recently, Kim et al. [39] found that ANGPTL4 may play a critical role in the regulation of insulin secretion. A limitation of our study is that we were, for technical reasons, unable to estimate insulin sensitivity in the dairy cows. The detailed metabolic mechanisms that underlie the action of ANGPTL4 on adipose tissue insulin sensitivity in cattle remain to be clarified.

Some studies have reported that hepatokines, especially FGF21, are involved in the regulation of hepatic lipid oxidation and ketogenesis during energy deprivation [40, 41]. However, the results of the present study showed that serum BHBA concentration did not appear to be associated with serum FGF21 and ANGPTL4 concentrations in dairy cows during the periparturient period. Serum BHBA concentration peaked at 1 wk postpartum, the timing of this peak was different from the peak in FGF21 and ANGPTL4 concentrations, which occurred on the day of parturition. Circulating levels of ketone bodies are determined by their rates of production and utilization. When the production of ketone bodies exceeds the extrahepatic tissue’s capacity to use them, they accumulate in the bloodstream, resulting in hyperketonemia [18]. Hence, we believe that the results of this study are not contradictory to the theory that FGF21 and ANGPTL4 are involved in the regulation of ketogenesis. Experimental studies have observed a positive linear correlation between hepatic FGF21 mRNA abundance and abundance in the hepatic mRNA for the gene encoding the key enzyme of ketogenesis in postpartum dairy cows [12]. That finding suggested that FGF21 could play a role in the regulation of ketogenesis in dairy cows during lactation, although there was no correlation between hepatic FGF21 mRNA abundance and concentration of BHBA in plasma. However, an earlier study conducted by Hotta et al. showed that blood BHBA levels were markedly increased in fasting FGF21 knock-out mice, indicating that FGF21 was not physiologically required for hepatic ketogenesis [42]. Further studies are needed to investigate the roles and precise mechanisms of hepatokines in ketogenesis in dairy cows.

NEB is considered as the etiological basis of energy metabolic disorders in periparturient dairy cows. Under NEB conditions, excessive fat mobilization results in a marked influx of NEFA in the blood; this influx is crucial to the etiology of energy metabolic disorders, such as fatty liver and ketosis. Fatty liver and ketosis are metabolic disorders that usually develop in dairy cows, particularly in high yielding dairy cows, during the periparturient period [43, 44]. Our results demonstrated that serum FGF21 and ANGPTL4 concentrations were significantly higher in cows with fatty liver and clinically ketotic cows compared with the healthy control cows. Studies have shown that energy insufficiency during early lactation in dairy cows was associated with increased plasma FGF21 [11]. In mice, hepatic expression and circulating levels of FGF21 were induced by both ketogenic diets and fasting [14]. Coincidentally, Loor et al. reported that expression of ANGPTL4 mRNA in the livers of feed-restricted and ketotic dairy cows was significantly higher than that observed in the healthy cows [13]. Moreover, our preliminary study showed that high concentrations of NEFA significantly promoted in vitro ANGPTL4 expression and secretion in bovine hepatocytes [15]. However, as far as we know, no relevant studies have assessed changes in serum ANGPTL4 levels in dairy cows with energy metabolism disorders. In addition, the exact mechanisms of FGF21 and ANGPTL4 on energy metabolism in dairy cows are still unclear. There is evidence that impairment of the insulin regulation of energy metabolism is considered to be an etiologic key factor for metabolic diseases [45]. According to our results, we speculate that upregulation of FGF21 and ANGPTL4 due to NEB could diminish adipose tissue insulin sensitivity and further aggravate lipolysis during early lactation in dairy cows, thus playing a role in the pathological processes of energy metabolism disorders.

In the present study, cows with fatty liver did not show any clinical symptoms of ketosis, and their serum BHBA concentrations were lower than in clinically ketotic cows. However, serum BHBA concentrations still were significantly higher in cows with fatty liver than in the control group. Thus, we could not rule out the possibility of the effects of BHBA on the expressions of FGF21 and ANGPTL4. In addition, this study was implemented in a commercial dairy farm. To avoid the stress and subsequent diseases (such as peritonitis and hepatapostema), liver biopsies were not performed on control group cattle. This is a potential flaw in our study. Although the choice of control group cows was based on the combination of veterinary clinical examination and blood biochemical analysis, the subjectivity of veterinarian diagnosis and the difference of metabolic state may still affect the results. Further studies are needed to clarify the effects of liver TAG content to the expressions of two hepatokines.

Here we showed that the serum activity of the enzymes AST, ALT and LDH were increased in cows with energy-related metabolic disorders, particularly in cows with fatty liver, which are consistent with the previous studies [46, 47]. Elevations in serum activity of these hepatic enzymes have been associated with the liver damage, although they are not ideal indicators for the diagnosis of liver disease in cattle because of they are not liver specific. TNF-α and IL-6 are major proinflammatory cytokines which play important roles in inflammatory response and lipid metabolism. TNF-α can promote IL-6 production and release by various pathways [48, 49]. There is strong evidence that TNF-α plays a role in fatty liver in rodent models. Moreover, serum TNF-α activity was increased in cows with fatty liver, and TNF-α can induce lipolysis by affecting insulin sensitivity [50, 51]. The result of this study showed that the serum concentrations of TNF-α and IL-6 also increased in cows with fatty liver, which further support inflammation as a component in the etiology of fatty liver.

## Conclusions

In summary, the present study showed that the serum FGF21 and ANGPTL4 concentrations change dramatically during the periparturient period in dairy cows, particularly during parturition and early lactation. Serum ANGPTL4 and FGF21 concentrations were positively correlated with serum concentration of NEFA in experimental cows during the periparturient period. Compared with healthy cows, cows with fatty liver and clinical ketosis exhibited significant higher serum concentrations of ANGPTL4 and FGF21. Although FGF21 and ANGPTL4 have important roles in the adaptation of energy metabolism, these two hepatokines may be involved in the pathological processes of energy metabolism disorders during the peripartum period in dairy cows.

## Methods

A commercial dairy farm located in Heilongjiang Province of China was selected to participate in the study.

### Selection of cows

The present study was divided into two experiments.

- *Experiment I*: Two hundred and nineteen periparturient Holstein cows were housed together in a free-stall barn and had ad libitum access to water. All cows were fed the same basal diet as a total mixed ration (TMR), which was formulated to meet the nutrient requirements recommended by the Dairy Cows Breeding Standard of China (2000, Table 2). The TMR was fed twice daily. TMR samples were collected weekly and composited monthly to measure the crude protein (Association of Official Analytical Chemists (AOAC), 2000; method 976.05), neutral detergent fiber (NDF), acid detergent fiber (ADF) [52], Ca and P (AOAC, 2000; method 935.13). In this experiment, the cows with subclinical or clinical hypocalcemia were excluded from the study. Based on the criteria from Roche and Berry, serum Ca levels of 2.0 and 1.4 mmol/L at calving day were proposed as thresholds of subclinical and clinical hypocalcemia, respectively [53].
- *Experment II*: Eighty-four cows of the same breed within 4 weeks after calving were provided by the commercial dairy farm. All cows were housed together in a free-stalls, fed the TMR twice a day, had ad libitum access to water, and milked twice daily. Individual cow milk yield were recorded by an electronic monitoring system daily throughout the experiment. Parity, body weight (BW), and milk production of the experimental cows are shown in Table 3. Currently, fatty liver can be diagnosed reliably only by performing biochemical analysis to determine the TAG content of a liver puncture biopsy sample (% wet weight). Cows with TAG contents > 10% in their livers were considered to suffer from fatty liver [6]. Cows with typical clinical signs of ketosis (such as anorexia, lethargy, constipation, loss of body weight, and decreased milk production) and serum BHBA concentrations > 1.2 mmol/L were considered to have clinical ketosis [51, 54]. A reference value for liver TAG contents of < 1% was used to exclude a diagnosis of fatty liver in the selected clinically ketotic cows. Cows with normal blood parameters (BHBA< 0.6 mmol/L, NEFA< 0.4 mmol/L) and no clinical signs of metabolic disorders were considered to be healthy controls. In the present study, 54 cows were liver biopsied in order to generate the group of cows with fatty liver. To narrow the screening process, general symptoms can be assessed to make a preliminary diagnosis, such as anorexia, depression, rapid loss of weight, and decreased milk yield. Meanwhile, serum BHBA concentrations < 1.2 mmol/L and no clinical signs of ketosis (including lethargy, constipation and nervous signs) were used to exclude a diagnosis of ketosis in the selected cows with fatty liver. Finally, 25 cows with fatty liver were selected according to the TAG contents of their livers. Twenty-nine cows with ketosis were chosen according to the serum BHBA concentration and TAG contents of their livers. Thirty cows were used to be healthy controls.

**Table 2:** Ingredient and nutrient composition of the antepartum and postpartum diets on a dry matter (DM) basis

Item	Antepartum	Postpartum
Ingredient		
Corn silage, %	38.1	30.9
Hay crop silage, %	12.0	15.5
Grass hay, %	12.6	–
Alfalfa hay, %	–	8.6
Ground shelled corn, %	21.1	24.6
Soybean meal (47.5% CP), %	4.0	3.7
Expeller soybean meal, %	5.6	7.7
Whole cottonseeds, %	3.6	5.6
Sodium bicarbonate, %	–	0.5
Mineral and vitamin mix, %	2.9	2.9
Energy and nutrients		
NE_L_, Mcal/kg	1.54	1.74
Crude protein (CP), %	15.6	16.4
MP supply, g/d	1433	2099
Neutral detergent fiber (NDF), %	35.7	34.3
Acid detergent fiber (ADF), %	23.4	22.0
Calcium (Ca), %	0.91	1.07
Phosphorus (P), %	0.45	0.49
Magnesium (Mg), %	0.46	0.32
Sodium (Na), %	0.14	0.13
Potassium (K), %	1.48	1.40
Chloride (Cl), %	0.76	0.39
Sulfur (S), %	0.20	0.22

**Table 3:** Parity, BW, and milk production of cows with clinical ketosis and fatty liver and healthy cows (means ± SE)

Item	Healthy (*n* = 30)	Clinical Ketosis (*n* = 29)	Fatty liver (*n* = 25)
Parity	3.73 ± 0.94	3.65 ± 0.71	3.58 ± 0.69
BW (kg)	661.89 ± 52.15	599.87 ± 71.54*	572.43 ± 66.23*
Milk production (kg/d)	31.13 ± 2.96	28.35 ± 3.23	26.44 ± 4.45

*P < 0.05 versus healthy cows

### Sampling and analyses

#### Blood

In the first part of the study, blood samples collected from the coccygeal vein in the early morning before feeding were obtained weekly from 4 wk antepartum to 4 wk postpartum. Within 1 h of collection, samples were carried to the laboratory, placed at room temperature for 30 min and centrifuged at 4000×*g* for 10 min to obtain serum. Sera were stored in sterile tubes at − 80 °C until further analysis.

Serum NEFA, BHBA, AST, ALT, LDH and ALP concentrations were assayed using corresponding kits (enzymatic method, Randox Laboratories Ltd., Ibach, Switzerland), respectively. All assays were analyzed using a Hitachi 7170 auto-analyzer (Hitachi Co., Tokyo, Japan). Serum ANGPTL4, FGF21, TNF-α and IL-6 levels were measured using commercial bovine ELISA kits (Cusabio Biotech Co. Ltd., Wuhan, China; ANGPTL4, Catalog No. CSB-EL001712BO; FGF21, Catalog No. CSB-EL008627BO; TNF-α, Catalog No. CSB-E12020B; IL-6, Catalog No. CSB-E12899B). All kits were used according to the manufacturer’s instructions.

#### Liver

In the second part of the study, liver tissue samples were collected by an experienced veterinarian using a liver biopsy needle (Shanghai Surgical Equipment Factory, Shanghai, China). Immediately after collection, liver tissue biopsies were rinsed with saline, frozen in liquid nitrogen, and stored at − 80 °C for determination of liver TAG content. Liver TAG content was measured after extraction according to Schlegen et al. [24]. In brief, lipids from liver biopsy specimens were extracted with a mixture of n-hexane and isopropanol (3:2, vol/vol). An aliquot of the extracts containing 25 to 50 nmol of TAG was pipetted into a glass vial (1.5 mL). After evaporation of the solvent by vacuum, the lipids were resolved in a 20 μL portion of a 1:1-mixture of chloroform and Triton X-100, and again, the solvent was evaporated. TAG content was measured by colorimetry using a commercially available enzymatic TAG kit (Applygen Technologies Inc., Beijing, China) and an automatic biochemical analyzer (Shenyang EKSV Medical Equipment Co., Ltd., China).

### Data analysis

Statistical analysis was performed using GraphPad Prism 5, version 5.01 (GraphPad Software Inc., USA). The normality distribution was tested by using the Kolmogorov-Smirnov (KS). Data about serum concentrations of ANGPTL4, FGF21, NEFA and BHBA in periparturient dairy cows, that were normally distributed, were analyzed using repeated-measures analysis of variance (ANOVA) followed by Bonferroni’s multiple comparison test. The estimated effects of sampling time on analyte levels were expressed as means ± standard errors (SE). The correlations of serum NEFA and BHBA concentrations and ANGPTL4 and FGF21 concentrations in experimental cows were analyzed by Pearson’s correlation analysis. Mean serum concentrations of ANGPTL4, FGF21, NEFA, BHBA, AST, ALT, LDH, ALP, TNFα and IL-6 in cows with clinical ketosis and fatty liver, and healthy cows (control group) were compared using one-way ANOVA followed by Dunnett’s multiple comparisons test. The results were expressed as the means ± SE. *P* < 0.05 was considered statistically significant, and *P* < 0.01 was considered highly statistically significant.

## Abbreviations

ALP: Alkaline phosphatase
ALT: Alanine aminotransferase
ANGPTL4: Angiopoietin-like protein 4
AST: Aspartate aminotransferase
BCS: Body condition scores
BHBA: β-hydroxybutyrate
FGF21: Fibroblast growth factor 21
IL-6: Interleukin-6
LDH: Lactic dehydrogenase
LPL: Lipoprotein lipase
NEB: Negative energy balance
NEFA: Non-esterified fatty acids
PPARα: Peroxisome proliferator-activated receptor α
TAG: Triacylglycerols
TNF-α: Tumor necrosis factor-α
